# Neonatal cardiac tamponade, a life-threatening complication secondary to peripherally inserted central catheter: a case report

**DOI:** 10.1186/s13256-022-03506-4

**Published:** 2022-07-28

**Authors:** Mohammad Reza Zarkesh, Mokaram Haghjoo

**Affiliations:** 1 Maternal, Fetal, and Neonatal Research Center, Family Health Institute, Tehran University of Medical Sciences, Sarv Ave., North Nejatolahi Street, Tehran, 1598718311 Iran; 2 Department of Neonatology, Yas Women Hospital, Tehran University of Medical Sciences, Tehran, Iran

**Keywords:** Neonate, Tamponade, Peripherally inserted central catheter

## Abstract

**Background:**

Although the use of a peripherally inserted central catheter (PICC) has many advantages for the treatment of neonates, catheter malposition may result in serious complications that could be life-threatening. We report the case of a 10-day-old neonate with cardiac tamponade secondary to a PICC line who was successfully treated by pericardiocentesis.

**Case presentation:**

An Iranian (Asian) preterm male neonate was born by Cesarean section with a birth weight of 1190 g and a first-minute Apgar score of 7. Based on an increased respiratory distress syndrome (RDS) score from 4 to 7, resuscitation measures and intubation were performed at the neonatal intensive care unit (NICU). On day 3 after birth, a PICC line was inserted for parenteral therapy. A chest X-ray confirmed that the tip of the PICC line was in the appropriate position. Mechanical ventilation was discontinued 72 h post-NICU admission because of the improved respiratory condition. On the day 10 post-NICU admission, he suddenly developed hypotonia, apnea, hypoxia, hypotension, and bradycardia. Resuscitation and ventilation support were immediately started, and inotropic drugs were also given. Emergency echocardiography showed a severe pericardial effusion with tamponade. The PICC line was removed, and urgent pericardiocentesis was carried out. The respiratory situation gradually improved, the O_2_ saturation increased to 95%, and vital signs remained stable.

**Conclusions:**

Dramatic improvement of the neonate's clinical responses after pericardial drainage and PICC removal were suggestive of PICC displacement, pericardial perforation, and cardiac tamponade.

**Supplementary Information:**

The online version contains supplementary material available at https://doi.org/10.1186/s13256-022-03506-4.

## Background

A peripherally inserted central catheter (PICC) made of silicone, polyurethane, or polyethylene provides a prolonged route for administration of parenteral fluids, nutrition, and medications. Preterm neonates admitted to hospital usually require a PICC because of their small and fragile vessels [1, 2]. Femoral, subclavian, and internal jugular veins are the most common sites used for PICC catheterization [3]. Although use of a PICC has many advantages for the treatment of neonates, catheter malposition may result in serious complications that could be life-threatening [4]. These potential complications (sepsis, embolism, intravascular thrombosis, pleural effusion, and pericardial effusion with tamponade) indicate that insertion of a PICC line requires special medical qualification and follow-up examination to ensure its proper location (the inferior third of the superior vena cava) [5–8]. Of the above complications, tamponade is a rare but fatal condition that occurs following catheter dislocation and pericardial perforation [8]. Cardiac tamponade is responsible for 47–100% of the mortality rate [9].

We report here the case of a neonate admitted to a neonatal intensive care unit (NICU) with pericardial effusion and cardiac tamponade secondary to a PICC line that was successfully treated with immediate PICC removal and pericardiocentesis.

## Case presentation

**Fig. 1:**
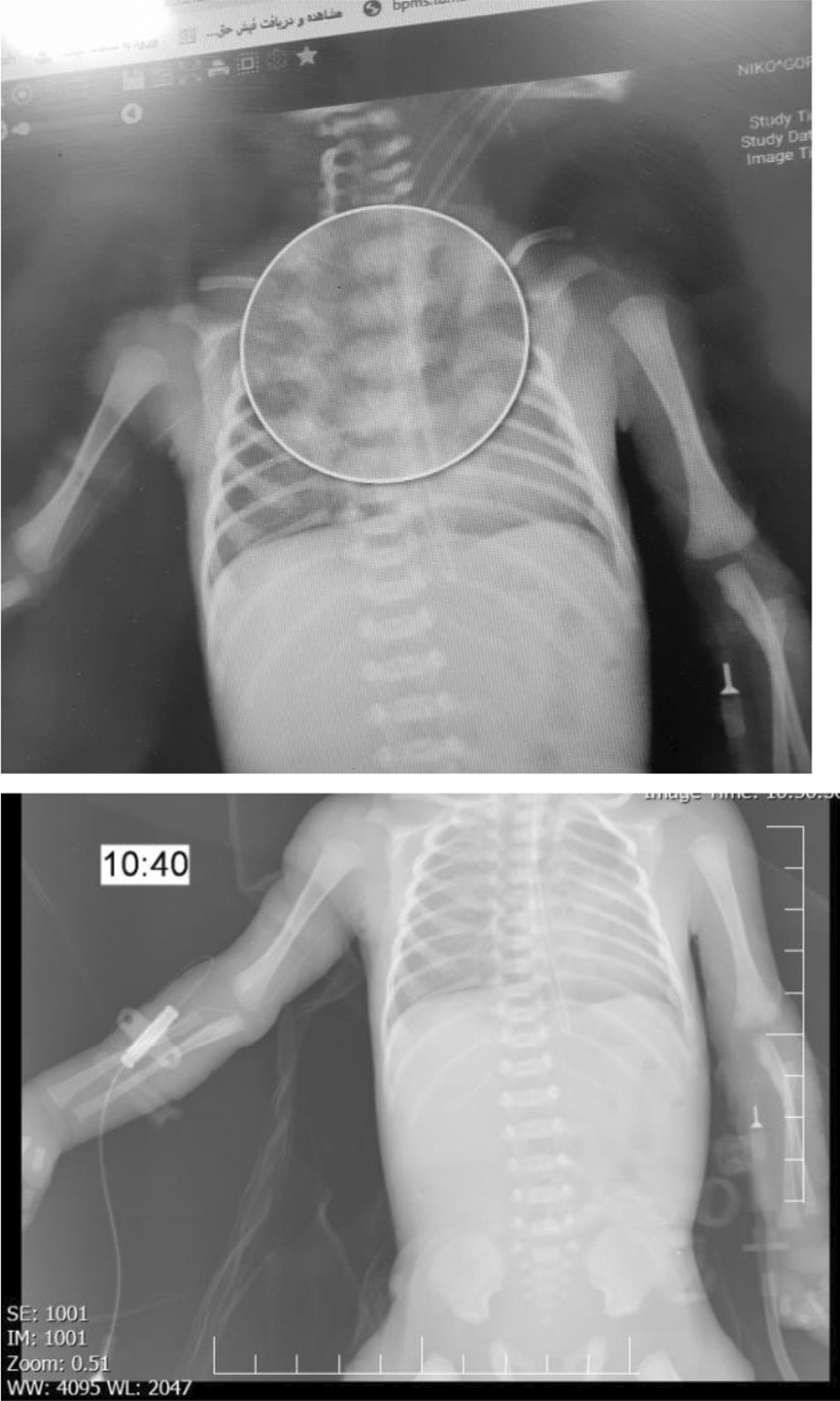
Chest X-ray findings showing the tip of the peripherally inserted central catheter line at appropriate position

An Iranian (Asian) pregnant women aged 32 years old with a primary complaint of preterm uterine contraction and vaginal bleeding presented the obstetric ward of Yas Hospital (Tehran-Iran, January 2021). She was a multi-para woman (Gravida: 3, Para: 2, Living: 2, Abortion: 0) with a gestational age of 30 weeks. She did not report any prenatal complications and had received corticosteroid therapy 2 days prior to hospital admission. Upon presentation, magnesium sulfate was immediately administered but she was transferred to the operation room for repeat Cesarean section due to progressive cervical dilation. A preterm male newborn with a first-minute Apgar score of 7 and birth weight of 1190 g was born. Physical examination after birth showed a notable frontal bossing, acrocyanosis, and diffuse head, neck, hand, and leg ecchymosis. Anterior and posterior fontanelles were normal. No congenital anomaly or abdominal organomegaly was observed. Peripheral pulses were palpable and heart sounds were normal without any abnormal sounds. His reflexes were normal, but limb movements were hypotonic. Due to an increase in his respiratory distress syndrome (RDS) score from 4 to 7 and signs of respiratory distress (inter- and subcostal retraction, grunting, and decreased breathing sounds), resuscitation measures were initiated. He was then given nasal continuous positive airway pressure (CPAP) in the operation room that was subsequently switched to mechanical ventilation in the NICU. Intratracheal surfactant and intravenous antibiotic therapy were also initiated in the NICU. Under mechanical ventilation with synchronized intermittent mandatory ventilation (SIMV) setup, his O_2_ saturation, blood pressure, and heart rate improved to > 94%, 70/35 mmHg, and 160 beats per minute, respectively. On day 3 post-NICU admission,, a PICC line (Premicath; 1Fr/28 G; VYGON, Écouen, France; Code 1261.080) was also inserted from the auxiliary vein of the right upper extremity for intravenous therapy, delivery of medications, and parenteral nutrition. A chest X-ray showed that the tip of the PICC line was in the appropriate place in the superior vena cava (SVC) (Fig. 1). Mechanical ventilation was discontinued 72 h post-NICU admission because of improved respiratory condition and normal automatic breathing. After extubation, low-flow oxygen therapy was continued and low-volume feeding was initiated based on his tolerance. His weight gain was acceptable and the clinical situation improved, including a gradual increase in the feeding volume and respiratory support. On day 10 post-NICU admission, he suddenly developed hypotonia, apnea, hypoxia, and bradycardia. He was gasping, had hypotension, and there was an absence of peripheral pulses. In the first minutes after the alarm signs, resuscitation was initiated and ventilation support provided, together with administration of inotropic drugs (1–3 minutes). Despite the high-frequency setup of the mechanical ventilator, the endotracheal tube was checked for displacement or obstruction due to his persistent critical condition (hypotension and shortness of breath). Pneumothorax and equipment failure were also ruled out. Several medical measures were initiated (approximately at minutes 3–4), including nothing by mouth (NPO), high-dose antibiotic therapy, emergency echocardiography, brain ultrasound, arterial and venous blood sampling, blood cell count, and arterial blood gas analysis.

**Fig. 2:**
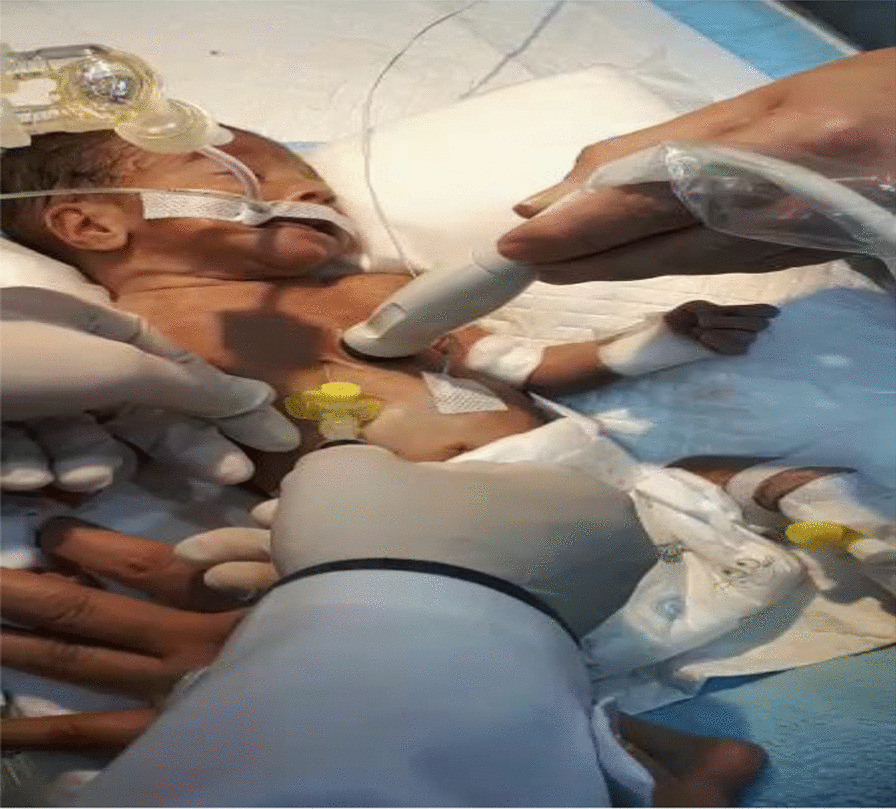
Emergency echocardiography

Echocardiography was performed over the next 5–7 minutes (Fig. 2), and the results showed SVC perforation caused by the catheter, severe pericardial effusion, right atrial collapse, ventricle dysfunction, and tamponade. The ejection fraction value was 40%, and there was no evidence of atrial septal defect (ASD), pleural effusion, or patent ductus arteriosus (PDA). A peripheral venous catheter was inserted, and the PICC was removed at the same time. Urgent pericardiocentesis was performed through subxiphoid cannulation guided by echocardiography and 10 ml of the pericardial fluid was aspirated (over the next 7–15 minutes) (Electronic Supplementary Material file 1: Video S1). The aspirated fluid was sent to the laboratory for tests, with the results showing severe acidosis (pH 6.80) and hyperglycemia (Table 1). To improve the hyperglycemia and metabolic acidosis, the patient was hydrated with low glucose solution and bicarbonate was initiated. Respiratory support was continued and antibiotics covering *Staphylococcus aureus* and Gram-negative bacteria were administered. Brain sonography was also performed, which was normal. The workup sequence is shown in Table 2.

**Table 1:** Results of arterial blood gas and laboratory blood tests

Factors	Before pericardiocentesis (range)	After pericardiocentesis (range)
Arterial blood gas results		
pH	6.8	7.37
Partial pressure of carbon dioxide (PmmHg)	24	34
Partial pressure of oxygen (mmHg)	55	106
O_2_ saturation (%)	–	98
HCO_3_ (meq/L)	–	19.7
Base excess (mEq/L)	–	− 50
Blood tests		
Hemoglobin (g/dL)	13	
Hematocrit (%)	42.7	
Platelet (cells/mcL)	150,000	
Mean corpuscular volume (fL)	122.3	
Mean corpuscular hemoglobin (pg)	37.2	
Mean corpuscular hemoglobin concentration (g/dL)	30.4	
White blood cells (cells/mcL)	14,200	
Neutrophils (cells/mcL)	42	
Eosinophils (cells/mcL)	3	
Monocytes (cells/mcL)	5	
Lymphocytes (cells/mcL)	50	
Creatinine (mg/mL)	0.7	
Natrium (mmol/L)	139	
Calcium (mg/dL)	9	
Potassium (mmol/L)	5.2	
Magnesium (mEq/L)	2.3	
C-reactive protein (mg/L)	1	
Blood sugar (mg/dl)	850	131
Throxine (pmol/L)	9.3	
Thyroid-stimulating hormone (mlU/L)	0.6	
Free thyroxine (pmol/L)	0.9	
Blood culture	Negative	Negative

**Table 2:** Sequence of performed workups following onset of alarms

Sequential steps	Measures	Time (minutes following first alarm signs)
1	Resuscitation, ventilation supports, administration of Inotropic drugs	1–3
2	Request for medical measures, including nothing by mouth, high-dose antibiotic therapy, emergency echocardiography, brain ultrasound examinations, and blood sampling	3–4
3	Echocardiography examination	5–7
4	Peripheral venous line catheterization, PICC removal, and pericardiocentesis	7–15
5	Hydration and bicarbonate infusion	15–20
6	Initiation of antibiotic therapy	20–23
7	Brain sonography	23-25

*PICC *Peripherally inserted central catheter

Under mechanical ventilation the respiratory parameters improved gradually; pericardial drainage and PICC removal occurred concurrently. O_2_ saturation increased to 95%, blood pressure elevated, and vital signs remained stable. Analysis of the aspirated pericardial fluid showed a hyperglycemic fluid (Table 3). A dramatic improvement in the neonate's clinical responses (after pericardial drainage and PICC removal) was suggestive of PICC displacement, pericardial perforation, and cardiac tamponade. Echocardiography was repeated (× 3) every 12 hours to ensure no further pericardial effusion was present. On day 40 after birth, the patient was still in the hospital but showed weight gain; he was in good condition without requiring respiratory support.

**Table 3:** Results of laboratory analysis of aspirated pericardial fluid

Factors	Range
Glucose (mg/dL)	2233
White blood cell (cells/mcL)	–
Red blood cell (cells/mcL)	300
Lactate dehydrogenase (units/L)	63
Protein (g/L)	108
Culture	Negative

## Discussion and conclusion

It is commone practice to insert PICC lines for parenteral administration in preterm neonates. Although PICC lines are associated with a number of catheter-related complications, such as infection, catheter block, catheter migration, thromboembolism, and catheter damage, pericardial effusion following PICC insertion is an unusual complication [10].

In the case presented here, we highlight the potential risk of pericardial effusion with tamponade as a fatal complication in a neonate 1 week after PICC insertion. Although this complication is rare and uncommon following PICC insertion (0.5–2%) [11], the diagnosis was suspected immediately in our case and confirmed by echocardiography. The patient recovered gradually after urgent removal of the PICC line followed by pericardiocentesis. Regarding the etiology of tamponade, we believe that the catheter tip eroded the catheterized vessel (SVC) and perforated the pericardium, allowing the infused fluid to move into the pericardial space. This flow of fluid into the pericardia space subsequently increased the pressure on the cardiac chambers, resulting in cardiac tamponade.

In accordance with our findings, da Silva Dornaus *et al*. also reported a preterm neonate (30 weeks) with cardiac tamponade secondary to PICC [8]. The neonate showed episodes of bradycardia, low O_2_ saturation, cyanosis, dyspnea, and worsening of clinical condition 5 days after PICC insertion. Radiography and echocardiography examinations revealed that the tip of the catheter was in the cardiac chamber with pericardial effusion and signs of tamponade. These authors also reported improvement in the neonate's clinical situation after immediate cardiac puncture and extraction of 25 mL of fluid similar to the infused solution [8].

An autopsy study by Warren* et al*. also showed pericardial effusion with tamponade in five neonates who died unexpectedly and suddenly after receiving parenteral nutrition via PICC. The autopsy findings showed endocardial injury and pericardial filling with permeation of a hyperosmotic parenteral fluid [12].

Iyer *et al*. reported cardiac tamponade in a 29-week-old preterm neonate with a sudden arrhythmia, unstable vital signs, and decreased O_2_ saturation. The neonate recovered after emergency intubation, administration of inotropic agents, and fluid aspiration from the pericardial space using an echocardiography-guided tap [10].

It should be noted that in cases with cardiac tamponade, the signs and symptoms may be non-specific and misleading (dyspnea, chest pain, tachycardia, hypotension, and non-palpable peripheral pulses). It is important that all infusions through the PICC must be stopped when tamponade is suspected. It has also been reported that in addition to catheter displacement, other factors, such as the material, length, and size of the catheter, duration of parenteral nutrition, osmolarity, and composition of the infused fluids may severely affect and worsen complications and outcomes related to cardiac tamponade. For example, it was reported in one study that pericardial presence of high-potassium infused fluid resulted in electrocardiogram alterations showing a pattern of hyperkalemia instead of a pattern of cardiac tamponade [1, 9, 12]. Another study also showed that tamponade was frequently observed in cases with PICC inserted through aperipheral vein compared to a central vein [4].

In conclusion, th case presented here highlights the potential risk of cardiac tamponade following central line insertion with sudden and unexpected symptoms associated with cardiovascular collapse. In such conditions, it is important to rapidly confirm that the catheter tip had migrated into the pericardial space and urgently initiate pericardiocentesis and catheter removal, all of which are effective measures to prevent death. Based on our and previous cases, we strongly recommend using catheters with soft tips, minimizing the movements of the neonates, and checking the catheter position periodically to reduce the risk of perforation and prevent life-threatening complications. Moreover, pericardial effusion with tamponade should be suspected in every case with PICC, and all neonatologists should be aware of this clinical emergency and the steps to be taken [1, 10]. Further studies are also needed to suggest other preventive strategies.

## Supplementary Information

**Additional file 1.** Video S1.

## Data Availability

The datasets are available from the corresponding author on reasonable request.

## Abbreviations

ASD: Atrial septum defect
CPAP: Continuous positive airway pressure
NICU: Neonatal intensive care unit
NPO: Nothing by mouth
PDA: Patent ductus arteriosus
PICC: Peripherally inserted central catheter
RDS: Respiratory distress syndrome
SIMV: Synchronized intermittent mandatory ventilation
SVC: Superior vena cava
